# Mutations of the *Cx43* Gene in Non-Small Cell Lung Cancer: Association with Aberrant Localization of Cx43 Protein Expression and Tumor Progression

**DOI:** 10.3390/medicina60101641

**Published:** 2024-10-07

**Authors:** Jung-Chien Chen, Kun-Tu Yeh, Yueh-Min Lin, Ya-Wen Cheng

**Affiliations:** 1 Graduate Institute of Cancer Biology and Drug Discovery, College of Medical Science and Technology, Taipei Medical University, Taipei 110, Taiwan; j8110100@seed.net.tw; 2 Department of General Surgery, Minimally invasive surgical center, Min-Sheng General Hospital, Taoyuan 330, Taiwan; 3 Ph.D. Program for Cancer Molecular Biology and Drug Discovery, College of Medical Science and Technology, Taipei Medical University and Academia Sinica, Taipei 110, Taiwan; 4 Department of Surgical Pathology, Changhua Christian Hospital, Changhua 500, Taiwan; 10159@cch.org.tw; 5 Department of Post-Baccalaureate Medicine, College of Medicine, National Chung Hsing University, Taichung 402, Taiwan; 6 Department of Pathology, Changhua Christian Hospital, Changhua 500, Taiwan; 93668@cch.org.tw; 7 School of Medicine, Chung Shan Medical University, Taichung 402, Taiwan; 8 Department of R&D, Stem Biotechnology Inc., 12F-2, Building F, No. 3, Park Street, Nangang District, Taipei 115, Taiwan

**Keywords:** *Cx43* gene mutation, lung cancer

## Abstract

*Background and Objectives*: The Connexin43 (*Cx43*) gene is a suspected tumor suppressor gene, as re-expressed wild-type *Cx43* genes reduce the malignancy potential of tumor cells. However, the role of *Cx43* gene expression in human lung tumorigenesis remains unclear. *Materials and Methods:* Tumor tissues from 165 primary lung cancer patients were collected to study Cx43 protein expression and gene mutations using immunohistochemistry and direct DNA sequencing. In addition, *Cx43* genes with or without mutations were transfected to CL-3 human lung cancer cells to confirm the function of these mutant forms of the *Cx43* gene. *Results:* Aberrant localization of Cx43 protein in the nucleus and cytoplasm of tumor cells was detected in 14 out of 165 non-small cell lung cancer (NSCLC) patients. Mutations in the *Cx43* gene were also found in patients with aberrant Cx43 localization, and transfection of these mutant genes into lung cancer cells enhanced their proliferation. *Conclusions:* To our knowledge, this is the first study to demonstrate *Cx43* gene mutations in human lung neoplasm, supporting the hypothesis that Cx43 may function as a tumor suppressor in some lung cancer patients. Additionally, the findings suggest an association between aberrant localization of Cx43 protein expression and tumor progression.

## 1. Introduction

Cx43 is a 43 kDa protein encoded by the *Cx43* gene, predominantly expressed in cardiac and smooth muscle, various epithelia, and endothelial, mesenchymal, and lung epithelial cells [1,2,3,4,5]. Recent studies have highlighted the potential role of Cx43 in cancer development and progression, particularly in lung cancer [6,7,8,9]. Multiple investigations have reported a significant reduction in Cx43 protein expression in lung tumors compared to non-tumor lung tissues [6,7,8]. This decreased expression is not unique to lung cancer but has also been observed in other malignancies, including prostate adenocarcinoma [10], mammary tumors [11,12], and breast cancer [4,12]. The consistent downregulation of Cx43 across various cancer types suggests its potential involvement in human malignancy.

The tumor suppressor function of Cx43 has been demonstrated through in vitro studies. Transfection of the *Cx43* gene into human lung carcinoma cell lines lacking Cx43 expression resulted in significantly reduced growth rates and decreased tumorigenicity compared to parent tumor cells [13]. Similar effects were observed in human glioblastoma cells [14,15]. Conversely, rat glioma cells transfected with mutant *Cx43* cDNA exhibited restored growth capacity and enhanced tumorigenicity [16]. These findings collectively support the hypothesis that Cx43 functions as a tumor suppressor gene [6,11,13,14,16,17,18,19,20].

The reduction in *Cx43* gene expression observed in various cancers may be attributed to abnormalities in transcription, post-translational modifications, or mutations in the *Cx43* gene [5,6,17]. However, mutations in connexin genes are relatively rare in human cancers. Studies on *Cx32* gene mutations in human liver and stomach tumors [19,21], as well as investigations of *Cx37* gene mutations in human breast carcinomas and lung adenocarcinomas [22], have yielded largely negative results. Despite this rarity, some evidence of connexin gene mutations has been reported in animal models, such as a mutation at codon 220 of *Cx32* in rat liver tumors [23] and mutations at codon 319 of *Cx37* in rat hemangiosarcomas [24]. Notably, Dubina et al. (2002) reported that mutational alterations of Cx43 are involved in advanced stages of human colon cancer progression [25]. This finding suggests that *Cx43* mutations may play a role in the malignant transformation of certain human cancers, including lung cancer.

The potential link between *Cx43* mutations and cancer progression, particularly in lung cancer, remains a relatively unexplored area. The aberrant localization of Cx43 protein observed in some cancer cells raises questions about the underlying genetic mechanisms [26,27,28]. We hypothesize that mutations in the *Cx43* gene may contribute to this aberrant protein localization and potentially influence tumor progression in NSCLC.

This study aims to address this knowledge gap by investigating *Cx43* gene mutations in NSCLC and examining their association with aberrant Cx43 protein localization and tumor progression. By focusing on NSCLC, a prevalent and aggressive form of lung cancer, we seek to elucidate the potential role of *Cx43* as a tumor suppressor gene in lung carcinogenesis. Our research aims to provide insights into the genetic basis of Cx43 dysfunction in NSCLC, potentially offering new perspectives on the molecular mechanisms underlying lung cancer progression. These findings could have important implications for understanding the pathogenesis of NSCLC and may open opportunities for novel diagnostic or therapeutic approaches targeting Cx43-related pathways.

## 2. Materials and Methods

### 2.1. Study Subjects

Lung tumor specimens were collected from 165 patients diagnosed with primary lung cancer. This cohort included 42 females and 123 males, all of whom were admitted to the Department of Thoracic Surgery at Taichung Veteran’s General Hospital (TVGH) in Taichung, Taiwan, between 1995 and 2003. Written informed consent was obtained from all participants in accordance with a biology study approved by the TVGH Institutional Review Board. None of the patients had undergone previous surgery or received any adjuvant therapy prior to the study. Following surgical resection, pathological material from various regions of each tumor was processed using conventional histological procedures. Fresh samples were immediately stored at −80 °C until further analysis. Tumors were classified based on the World Health Organization (WHO) criteria and staged according to the tumor-node-metastasis (TNM) system. Patients were monitored for survival data for up to 3000 days after surgery. Survival data were obtained from hospital records and through periodic interviews with patients and their relatives.

### 2.2. Immunohistochemistry (IHC)

All specimens were formalin fixed and paraffin embedded. Briefly, 3 µm sections were cut, mounted on glass slides, and dried overnight at 37 °C. All sections were then deparaffinized in xylene, rehydrated with alcohol, and washed in phosphate-buffered saline (PBS). This buffer was used for all subsequent washes. Sections for Cx43 detection were heated in a microwave oven twice for 5 min in citrate buffer (pH 6.0). A monoclonal anti-human Cx43 antibody (Zymed Laboratories, Inc., San Francisco, CA, USA, dilution 1:250) was used, with an incubation time of 60 min at room temperature. The conventional streptavidin peroxidase method (DAKO, LSAB Kit K675, Glostrup, Denmark) was performed with the Cx43 antibody. Slides were developed with 3,3′-diaminobenzidine for 5 min and counterstained with hematoxylin. Negative controls were obtained by excluding the primary antibody. The results were evaluated independently by three observers and scored for negative staining (0–25% positive), with cases having more than 25% positive staining regarded as positive.

### 2.3. Protein Extraction and Western Blotting

Total protein extracts from fresh lung tumor tissues were prepared with a lysis buffer (100 mM Tris, pH 8.0, 1% SDS). Protein concentration was determined using a Bio-Rad protein assay kit. The proteins were separated by SDS-PAGE on a 12.5% gel (1.5 mm thick). Following electrophoresis, proteins were transferred to Hybond-C extra nitrocellulose membranes. The membranes were then blocked with 5% nonfat milk in TBST. Cx43 protein detection was performed by incubating the membranes with the same anti-Cx43 antibody used in the IHC studies. After extensive washing with TBST, the membranes were incubated with a peroxidase-conjugated secondary antibody (1:500 dilution). The Cx43 band was visualized using enhanced chemiluminescence (NEN Life Science Products, Inc., Boston, MA, USA).

### 2.4. Preparation of DNA and RNA, Reverse Transcription Polymerase Chain Reaction (RT-PCR), and DNA Sequence Analysis

Total tumor RNA was extracted by homogenizing the tissues in 1 mL TRIzol reagent, followed by chloroform re-extraction and isopropanol precipitation. cDNA synthesis was performed in a total volume of 20 µL of reaction mixture containing 5 µg of total RNA in the RT reaction buffer (Gibco-BRL, Gaithersburg, MD, USA), 10 mM DTT, 100 pmole/mL of oligo (dT), 0.5 mM each of dNTP, and 200 units of M-MLV reverse transcriptase (Gibco-BRL). Samples were incubated at 42 °C for 50 min, and the reaction was terminated by heating the samples at 75 °C for 10 min, followed by quick chilling on ice. Target sequences were amplified in a 50 µL reaction mixture containing 20 pmol each of primers Cx43-S (5′-AGTGGTGACTGGAGCGCC-3′) and Cx43-AS (5′-CTAGATCTCCAGGTCATC-3′) for *Cx43*, 2.5 units of Taq polymerase (TAKARA Shuzo, Shiga, Tokyo, Japan), 0.5 mM dNTP, 5 µL PCR reaction buffer, and 1 µL cDNA. An initial denaturation cycle was performed for 5 min at 94 °C, followed by 35 cycles each for 40 s at 94 °C, 1 min at 54 °C, and 1 min 30 s at 72 °C. The PCR products were analyzed by 1% agarose gel electrophoresis. The DNA sequence of *Cx43* cDNA was analyzed using an autosequencing machine (Applied Biosystems 3100 Avant Genetic Analyzer; Applied Biosystems, Foster City, CA, USA) according to the manufacturer’s protocol.

To confirm the *Cx43* mutation, genomic DNA from the tumor tissues was prepared using proteinase K digestion, phenol-chloroform extraction, and ethanol precipitation for PCR amplification. Target sequences were amplified in a 50 µL reaction mixture containing 20 pmol of each primer, 2.5 units of Taq polymerase (TAKARA Shuzo, Shiga), 0.5 mM dNTP, 5 µL PCR reaction buffer, and 1 µL genomic DNA. The sequences of the primers were as follows:

(1) Cx43-1: Cx43-S (5′-AGTGGTGACTGGAGCGCC-3′) and Cx43-AS (5′-CTAGATCTCCAGGTCATC-3′);
(2) Cx43-2: Cx43-S (5′-CTAGATCTCCAGGTCATC-3′) and Cx43-AS (5′CTCGAGCTAGATCTCCAGGTCATC-3′). An initial denaturation cycle was performed for 5 min at 94 °C, followed by 35 cycles of 40 s at 94 °C, 40 s at 54 °C, and 1 min at 72 °C. The DNA sequence of the two PCR products from each patient was also analyzed by autosequencing.

### 2.5. Cell Culture and Cx43 Gene Transfection

The full-length wild-type and mutant *Cx43* genes were PCR-amplified from normal lung epithelial cells and lung tumor tissues and verified by direct sequencing. The resulting PCR products were purified using the GENECLEAN III kit and ligated into the pcDNA3.1/V5-His TOPO TA Expression Kit (Invitrogen, CA, USA) eukaryotic expression vector. The ligated DNA was used to transform competent JM109 Escherichia coli bacteria. Desired recombinants were selected by using ampicillin; then, the recombinant DNA was prepared in a large quantity and purified using the PlasPrep Kit (Blossom, Taipei, Taiwan). The calcium phosphate precipitation method was used to introduce those prepared recombinant DNA into the lung cancer cell line. For each transfection, 1 × 10^5^ cultured CL-3 cells per well were seeded the previous day. After a 12 h incubation, the cells, at 30–50% confluent, were washed twice with phenol red-free RPMI1640 medium without FBS. The cells were then incubated with 1 mL phenol red-free RPMI1640 medium with 10% FBS for 3 h. Subsequently, a calcium chloride-Hepes-buffer saline and recombinant DNA solution were added dropwise to the medium in each well, and the cells were returned to the incubator. After 4 h, the medium was aspirated, and the cells were shocked with a glycerol solution for 30 s and washed twice with PBS. Stable transfectants were selected by culturing the transfected cells in the medium containing the antibiotic G418. Cells were seeded at a density of 10^3^ cells per mL in 35 mm dishes and cultured for 24, 48, 72, 96, 120, and 144 h. Cell numbers were counted daily for 7 days (168 h), with measurements taken at 24 h intervals.

### 2.6. Statistical Analysis

Statistical analysis was performed using the SPSS statistical software program11.0 (SPSS Inc., Chicago, IL, USA). The associations between Cx43 expression and prognostic factors for lung cancer (age, sex, T, N, M, tumor stage, tumor type, tumor grade) were analyzed using the Pearson Chi-square test, Fisher’s exact test, and Likelihood ratio test. Survival curves were estimated using the Kaplan–Meier method, and survival rates were compared using the log-rank test.

## 3. Results

### 3.1. Aberrant Localization of Cx43 Protein Expression and Its Association with Tumor Progression in NSCLC

We analyzed Cx43 protein expression in 165 NSCLC samples using IHC. The demographic distribution showed a higher proportion of male patients (Table 1). This gender imbalance was adjusted for in the subsequent statistical analyses.

**Figure 1:**
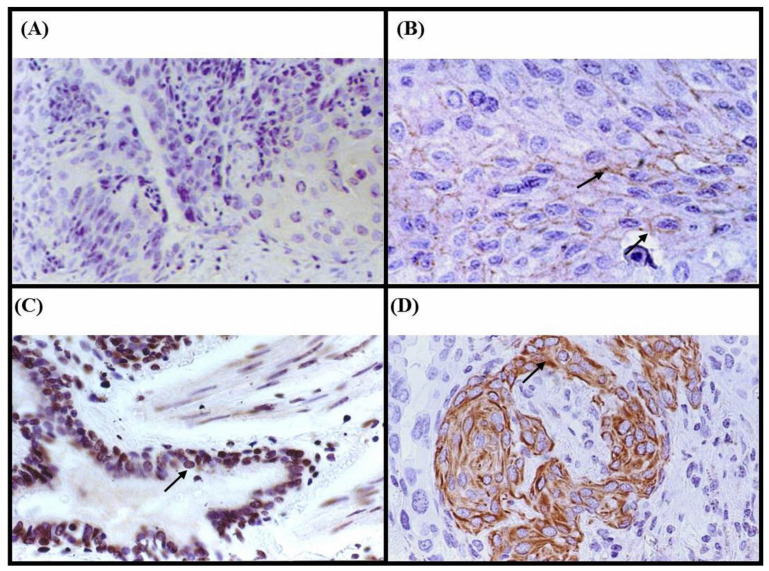
Immunohistochemical analysis of Cx43 protein expression using a monoclonal Cx43 anti-human antibody on paraffin sections of lung tumor specimens. (**A**) Negative control showing no Cx43 immunostaining (×200). (**B**) Positive control showing Cx43 protein expressed in the membrane of non-tumor lung tissue (arrow) (×400). (**C**) Nuclear localization of Cx43 in tumor cells (arrow) (patient 888C) (×400). (**D**) Cx43 expression in the cytoplasm of tumor cells (arrow) (patient 245D) (×400).

In normal lung tissue, Cx43 protein was consistently localized to the plasma membrane (Figure 1B), serving as a baseline for comparison. Among the 165 NSCLC samples, 34 (20.6%) exhibited Cx43 protein expression, with 14 (8.5%) showing aberrant localization. Specifically, 20 patients (12.1%) displayed plasma membrane localization (Figure 1B), similar to normal tissue. However, four patients (2.4%) showed unexpected nuclear localization (Figure 1C), and 10 patients (6.1%) exhibited cytoplasmic localization (Figure 1D), both representing aberrant patterns. The remaining 131 patients (79.4%) showed no detectable Cx43 expression, indicating a potential loss of function in these cases.

To validate and strengthen the IHC results, we performed Western blot analysis on tumor tissues from all 165 patients. This quantitative assessment provided a more sensitive measure of protein expression. The analysis confirmed Cx43 protein expression in all 34 IHC-positive samples, demonstrating perfect concordance between the two methods for positive cases. Interestingly, 27 of the 131 IHC-negative samples (20.6%) showed significantly reduced Cx43 expression by Western blot, suggesting that IHC may underestimate very low levels of expression. Overall, these findings confirmed that IHC accurately reflects Cx43 protein expression in fresh lung tumor tissues, with Western blot offering complementary sensitivity for borderline cases.

We conducted a comprehensive examination of associations between Cx43 expression and various clinicopathological factors (Table 1). Statistical analysis revealed that only the T factor was significantly associated with Cx43 protein expression (*p* = 0.046). Notably, aberrant Cx43 localization was more frequently observed in T3 and T4 tumors compared to T1 and T2 tumors, suggesting a potential link between aberrant Cx43 localization and tumor invasion complexity.

Conversely, no significant associations were found between Cx43 expression and other clinicopathological parameters. These included age (*p* = 0.468), gender (*p* = 0.117), tumor type (adenocarcinoma vs. squamous cell carcinoma, *p* = 0.571), overall tumor stage (*p* = 0.106), N factor (*p* = 0.130), and smoking status (*p* = 0.185). The lack of correlation with these factors suggests that Cx43 expression patterns may be independent of these conventional prognostic indicators.

These results collectively suggest that aberrant localization of Cx43 protein is specifically associated with more advanced T stage, potentially indicating a role in tumor progression and local invasion. The lack of significant associations with other clinicopathological factors implies that Cx43 expression may serve as an independent marker of tumor invasiveness in NSCLC. This finding opens up new opportunities for understanding the molecular mechanisms underlying NSCLC progression and may have implications for targeted therapies and prognostic assessments in the future.

### 3.2. Mutations in the Cx43 Gene and Their Association with Aberrant Localization of Cx43 Protein Expression in NSCLC

To investigate the potential link between aberrant localization of Cx43 protein expression in NSCLC and mutations in the *Cx43* gene, we conducted a comprehensive analysis of *Cx43* cDNA and genomic DNA samples. *Cx43* cDNA samples from 14 patients exhibiting aberrant protein localization were amplified by RT-PCR and subjected to direct sequencing. To confirm the presence of *Cx43* gene mutations, we also analyzed genomic DNA samples from both tumors and apparent non-tumor tissues using PCR amplification followed by direct sequencing.

Our analysis revealed *Cx43* gene mutations in 6 out of 14 patients (43.0%) with aberrant protein localization. In contrast, only 1 out of 151 patients (0.7%) with Cx43 plasma membrane expression or negative immunostaining exhibited a *Cx43* gene mutation. The specific mutation sites for these seven patients are shown in Table 2.

Among the identified mutation, three were silent mutations, while the remaining four were missense mutations. Notably, certain mutation sites, such as codons 18 and 215, were observed in multiple patients. Transversion mutations were more prevalent (5/7; 71.4%) compared to transition mutations (2/7, 28.6%). Analysis of the sequences surrounding the mutation sites revealed that five out of seven mutations occurred either at A:T base pairs or in close proximity to repetitive sequences and/or tandem repeat sequences. The identified mutations were primarily located in the N-terminal, extracellular loop, and membrane-spanning regions of the Cx43 protein, which may have implications for protein function and localization.

Regarding the clinical relevance of these findings, we observed that among the 14 patients with aberrant Cx43 immunostaining localization, four out of six patients (66.7%) harboring *Cx43* gene mutations presented with advanced tumor stages (IIIa or IIIb). This frequency was higher compared to patients without *Cx43* gene mutations, where only three out of eight (37.5%) had advanced tumor stages.

Table 2 provides a comprehensive overview of the *Cx43* DNA sequencing results for 15 patients, including those with Cx43 positive and/or negative immunostaining, along with their clinicopathological characteristics. The table details patient information, tumor characteristics, mutation sites, and corresponding amino acid changes.

Our findings suggest that *Cx43* gene mutations may partially account for the aberrant localization of Cx43 protein expression in NSCLC. Furthermore, these mutations were frequently observed in advanced-stage lung cancer patients exhibiting aberrant Cx43 immunostaining localization. These results provide valuable insights into the molecular mechanisms underlying Cx43 protein mislocalization in NSCLC and its potential association with disease progression.

### 3.3. Impact of Cx43 Mutations on Protein Localization and Cell Growth in NSCLC

To investigate the relationship between Cx43 mutations and cell growth in lung cancer, we conducted a series of mutant expression experiments. Our investigations revealed that transfection of mutant *Cx43* resulted in aberrant protein localization, while transfection of wild-type *Cx43* did not yield this effect (Supplementary Data).

**Figure 2:**
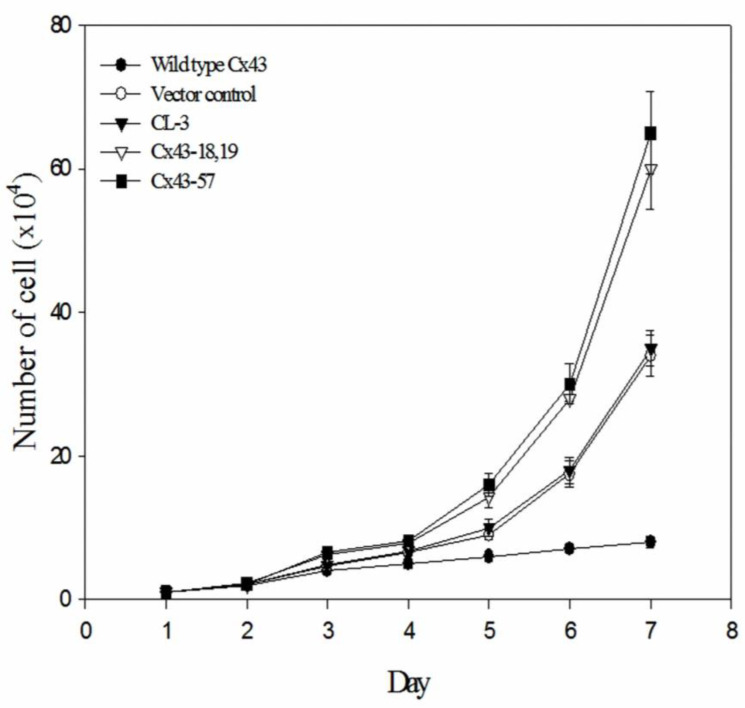
Growth curves of CL-3 lung cancer cells expressing wild-type or mutant Cx43. Cells were transfected with wild-type *Cx43*, vector control, or mutant *Cx43* constructs (Cx43-18,19 and Cx43-57). Cell numbers were counted daily for 7 days. Data points represent the mean of three independent experiments + standard error of the mean.

Figure 2 compares the proliferation rates of CL-3 lung adenocarcinoma cells expressing wild-type *Cx43*, vector control, parental CL-3 cells, and two mutant *Cx43* constructs (Cx43-18,19 and Cx43-57) over a 7-day period. Notably, cells expressing mutant Cx43 (Cx43-18,19 and Cx43-57) demonstrated significantly enhanced proliferation compared to those expressing wild-type Cx43, vector control, or the parental CL-3 cells. The growth curves reveal a clear divergence in cell proliferation patterns beginning around day 3. While the wild-type Cx43, vector control, and parental CL-3 cells maintained relatively modest growth rates, the mutant Cx43-expressing cells exhibited an obvious increase in cell numbers. This accelerated growth became increasingly distinct over time, with the difference most obvious by day 7.

Quantitatively, by the end of the 7-day observation period, the number of cells in the mutant Cx43 groups was approximately twice that of the control groups. These findings suggest that the expression of mutant Cx43 proteins in transfected lung tumor cells not only leads to aberrant localization of the protein but also promotes accelerated tumor cell growth. The substantial difference in proliferation rates between mutant and wild-type Cx43-expressing cells indicates that these mutations may contribute to a significant growth advantage in NSCLC cells.

Our results provide strong evidence that *Cx43* mutations alter both protein localization and cellular growth dynamics in NSCLC. This disruption of normal protein distribution, combined with increased proliferation, may contribute to the aggressive behavior of lung cancers with aberrant Cx43 localization.

### 3.4. Association of Cx43 Gene Mutations and Patient Survival in NSCLC

**Figure 3:**
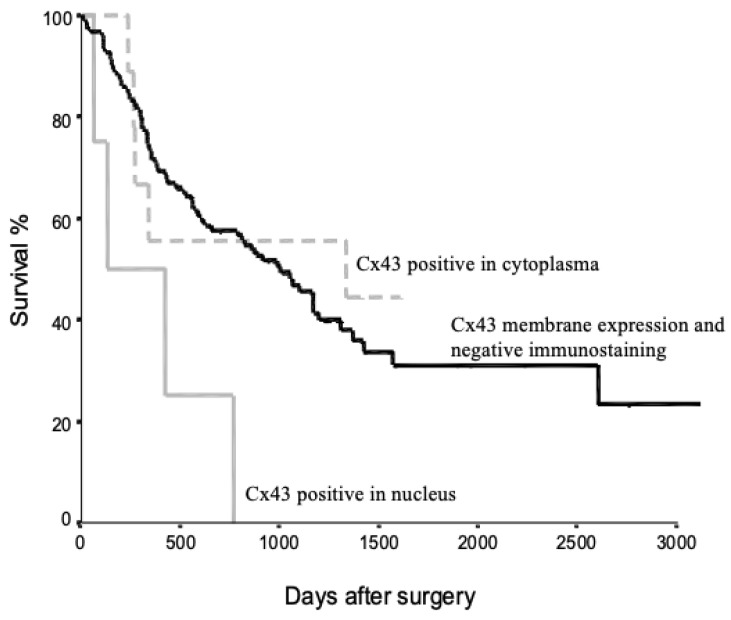
Kaplan–Meier survival curves for NSCLC patients stratified by Cx43 protein localization. Three groups are represented: patients with Cx43 membrane expression and negative immunostaining (black line), patients with Cx43 positive staining in the cytoplasm (dotted line), and patients with Cx43 positive staining in the nucleus (grey line). The *x*-axis represents days after surgery, while the *y*-axis shows the survival percentage.

Figure 3 presents the Kaplan–Meier survival analysis of NSCLC patients based on Cx43 protein localization. The survival curves demonstrate distinct outcomes for three patient groups: those with Cx43 membrane expression and negative immunostaining (black line), those with Cx43 positive staining in the cytoplasm (dotted line), and those with Cx43 positive staining in the nucleus (grey line).

Patients with Cx43 membrane expression and negative immunostaining exhibited the highest survival rates. In contrast, patients with Cx43 positive staining in the nucleus showed the poorest survival outcomes. The group with cytoplasmic Cx43 staining displayed an intermediate survival profile. These findings suggest a potential correlation between Cx43 localization and patient prognosis in NSCLC. The observed differences in survival curves indicate that aberrant Cx43 localization, particularly nuclear accumulation, may be associated with more aggressive disease progression and reduced overall survival.

These results collectively suggest that Cx43 mutations in NSCLC may lead to both aberrant protein localization and enhanced tumor cell proliferation, potentially affecting patient survival. Further statistical analysis would be necessary to determine the significance of the observed survival differences and to quantify the impact of *Cx43* mutations on long-term patient outcomes.

## 4. Discussion

This study provides the first evidence of *Cx43* mutation in NSCLC, revealing a novel association between these mutations and aberrant localization of Cx43 protein expression. Our findings demonstrate a significant difference in mutation frequency between patients with normal Cx43 localization or negative immunostaining (1/131, 0.7%) and those with aberrant Cx43 immunostaining (6/14, 42.9%). This remarkable difference suggests a potential causal relationship between Cx43 mutations and abnormal protein localization in NSCLC.

Connexin mutations have been previously implicated in various hereditary and somatic human diseases, such as X-linked Charcot-Marie-tooth disease (Cx32) and visceroatrial heterotaxia (Cx43) [29,30]. However, until now, connexin gene mutations have been rarely reported in human tumors [19,21,22], with only one study indicating *Cx43* mutational alterations in advanced stages of human colon cancer [25]. Our study detected *Cx43* gene mutations in 4.2% (7/165) of NSCLC patients, a finding that expands our understanding of the potential role of Cx43 in human neoplasms. The higher frequency of these mutations in advanced lung tumors, particularly those with aberrant Cx43 localization, suggests a possible association between *Cx43* gene mutations and lung tumor progression. These results are consistent with our immunohistochemical and Western blot analyses, which revealed aberrant Cx43 localization in 8.5% of NSCLC samples. The significant association between aberrant Cx43 localization and advanced T stage (T3 and T4) further supports the hypothesis that *Cx43* mutations may contribute to tumor invasion and progression in NSCLC.

This study significantly expands our understanding of *Cx43* gene expression and mutation in NSCLC. Our findings reveal a complex picture of Cx43 dysregulation, both expression changes and genetic alterations, in lung tumors. We observed positive Cx43 immunostaining in 8.5% (14/165; only N/C) of NSCLC samples, specifically in the nucleus or cytoplasm, consistent with aberrant localization. This observation aligns with previous research by Ruch et al. [31], who reported significantly reduced *Cx43* mRNA and protein levels across various lung cancer cell lines compared to non-transformed lung epithelial cells. Our data further corroborate these findings, demonstrating lower *Cx43* mRNA and protein levels in both tumor and non-tumorous lung tissue from NSCLC patients compared to non-cancer controls [20].

The aberrant localization of Cx43 protein in the cytoplasm and nuclei of tumor cells, as observed in our study, is consistent with previous reports [31]. This mislocalization, together with the overall reduction in Cx43 expression, suggests a profound disruption of Cx43 function in NSCLC. Our mutational analysis revealed *Cx43* gene mutations in 43.0% of patients with aberrant protein localization, compared to only 0.7% in patients with normal localization or negative immunostaining. This remarkable difference suggests that *Cx43* gene mutations may partially account for the aberrant localization of Cx43 protein in NSCLC. Notably, we observed a higher frequency of transversion mutations (71.4%) compared to transition mutations, with several mutations occurring near repetitive or tandem repeat sequences. The clinical relevance of these findings is underscored by the observation that 66.7% of patients with *Cx43* gene mutations and aberrant protein localization presented with advanced tumor stages (IIIa or IIIb) compared to 37.5% in patients without mutations. This association suggests a potential link between *Cx43* mutations, protein mislocalization, and disease progression in NSCLC.

The mechanisms underlying Cx43 dysregulation in lung cancer appear to be complex. While our study focuses on genetic mutations, epigenetic factors like promoter methylation may also play a role. Previous research has shown Cx43 promoter methylation in rat liver cells [32], and our earlier work detected promoter methylation in 64% of *Cx43* mRNA non-detectable tumors [20]. Similar epigenetic silencing has been observed for Cx26 in lung cancer [33]. These findings collectively suggest that both genetic mutations and epigenetic modifications contribute to the altered expression and localization of Cx43 in NSCLC, potentially impacting tumor progression and patient outcomes.

Previous studies have shown that post-translational modification, particularly phosphorylation by kinases such as protein kinase C (PKC), can affect Cx43 protein stability and expression [34,35]. Our preliminary observations suggested an association between increased PKC protein levels and aberrant Cx43 protein expression. This potential link is supported by the recent literature. Viczenczova et al. (2016) demonstrated that PKC signaling is involved in Cx43 regulation in cardiac tissue under stress conditions [36]. Furthermore, Pun et al. (2023) highlighted the critical role of PKC-mediated phosphorylation of Cx43 at Serine-368 in cardiac function and disease [37]. These studies support our hypothesis that Cx43 phosphorylation by PKC may be partially linked to Cx43 aberrant localization in lung tumorigenesis. Furthermore, our findings reveal an association between aberrant Cx43 protein localization and tumor size in NSCLC patients (Table 1). This observation aligns with previous reports of reduced *Cx43* mRNA and protein expression in lung carcinoma [31], suggesting that aberrant Cx43 localization may enhance malignancy in NSCLC.

Our study provides convincing evidence for the role of *Cx43* gene mutations in the aberrant localization of Cx43 protein in NSCLC. While *Cx43* gene expression levels correlate well with gap junction intercellular communication (GJIC) function in lung carcinoma cell lines, our findings suggest that Cx43 may contribute to tumorigenesis through mechanisms independent of GJIC suppression. Our study identified several mutation codons in the Cx43 protein, primarily in the N-terminal, extracellular loop, and membrane-spanning regions. These mutations appear to have significant functional consequences. Our transfection experiments demonstrated that mutant *Cx43* expression in CL-3 lung cancer cell lines resulted in aberrant protein localization, whereas wild-type *Cx43* transfection did not produce such effects (Supplementary Data). This observation aligns with previous studies showing that certain *Cx43* mutations can prevent plasma membrane localization or enhance tumorigenic potential [16,26,38].

Importantly, our proliferation assays revealed that cells expressing mutant Cx43 (Cx43-18,19 and Cx43-57) exhibited significantly enhanced proliferation compared to those expressing wild-type Cx43, vector control, or parental CL-3 cells. This accelerated growth became increasingly differentiated over time, with mutant Cx43-expressing cells showing approximately twice the cell numbers of control groups by day 7. These findings suggest that Cx43 mutations not only affect protein localization but also promote tumor cell growth, potentially contributing to the aggressive behavior of NSCLC.

The subcellular localization of Cx43 appears to play a crucial role in tumor growth regulation [39]. Typically, connexins are localized in the cell membrane with a punctuated expression pattern [2], and aberrant localization may contribute to the loss of intercellular communication via gap junctions [21]. Our survival analysis revealed that NSCLC patients with Cx43-positive immunostaining in the nucleus had significantly shorter survival rates compared to those with Cx43-negative immunostaining or cytoplasmic immunostaining. This observation suggests that Cx43 localized in the nucleus, cytoplasm and plasma membrane may play distinct roles in malignant and normal cells. These findings are consistent with previous studies showing that *Cx43* cDNA transfection can influence tumorigenicity in various cell types [16,35]. The effects of Cx43 on cell proliferation may be mediated through alterations in the expression of cell cycle regulatory genes [11,13,40,41]. Our results extend these observations to NSCLC, highlighting the potential of Cx43 as a tumor suppressor in some lung cancer patients.

In conclusion, our study provides the first evidence of *Cx43* gene mutations in human lung cancer and their association with aberrant localization and enhanced cell proliferation. The correlation between nuclear Cx43 localization and poor prognosis in NSCLC patients further underscores the clinical relevance of these findings. While our results support the hypothesis that the *Cx43* gene may act as a tumor suppressor in some lung cancer patients, further studies are required to elucidate the precise functional role of Cx43 in lung tumorigenesis signaling pathways. Future research should focus on characterizing the specific mechanisms by which Cx43 mutations and aberrant localization contribute to NSCLC progression. Additionally, investigating the potential of Cx43 as a prognostic marker or therapeutic target in NSCLC could provide valuable insights for clinical management.

## 5. Conclusions

In conclusion, this study provides significant evidence demonstrating the presence of *Cx43* gene mutations in NSCLC patients, highlighting an important association with aberrant localization of Cx43 protein expression. Our findings reveal that *Cx43* gene mutations may be partially associated with the abnormal localization of Cx43 protein in the cytoplasm and/or nucleus of lung tumors. Importantly, we demonstrated that NSCLC patients with aberrant nuclear localization of Cx43 protein have a worse prognosis compared to patients with Cx43-negative and membrane-localized Cx43 protein. This observation supports the hypothesis that properly localized Cx43 may act as a tumor suppressor in some lung cancer patients. The identification of *Cx43* gene mutations in a subset of lung cancer patients with abnormal protein localization further strengthens this hypothesis. As this study marks the first report of *Cx43* gene mutations in human lung cancer, our results highlight the importance of further investigations to elucidate the functional implications of Cx43 mutations in lung tumorigenesis, their impact on protein localization, and their influence on patient outcomes.

## Figures and Tables

**Table 1:** Relationships between Cx43 protein expression and clinicopathological characteristics.

Clinical Parameters		No. of Patients	Cx43 IHC			*p*
			−	+ (Membrane)	+ (N/C)	
Total		165	131	20	14	
Sex						
	Female	42	41		1	0.117
	Male	123	110		13	
Age (years)						
	<55	28	27		1	0.468
	>55	137	124		13	
Tumor type						
	AD	98	91		7	0.571
	SQ	67	60		7	
Tumor stage						
	I	69	66		3	0.106
	II and III	96	85		11	
T factor						
	T1 and T2	129	121		8	0.046
	T3 and T4	36	30		6	
N factor						
	No	88	80		8	0.130
	N1	35	30		5	
	N2	42	41		1	
Smoking status						
	Non-smoker	75	71		4	0.185
	Smoker	90	80		10	

AD: adenocarcinoma; SQ: squamous cell carcinoma; IHC: Immunohistochemistry; N/C: Nuclear/Cytoplasmic. Statistical analysis was performed using the Pearson Chi-square test or Fisher’s exact test, as appropriate. A *p*-value less than 0.05 is considered statistically significant. Note: “−” indicates negative staining, and “+(N/C)” indicates positive nuclear or cytoplasmic staining.

**Table 2:** *Cx43* DNA sequencing results of 15 patients with Cx43 positive and/or negative immunostaining and their clinicopathological characteristic.

Patient No.	Sex	Age	Smoking *	Location **	Tumor		T	N	Mutation Site			Amino Acid Change
					Type ***	Stage			Codon	Type	Surrounding Sequence	
245D	M	66	1.10	C	SQ	IIIa	3	1	61	T.A/C.G	GGTTGTGAA	Cys->Cys
964E	M	68	4.38	C	SQ	I	2	0	18	A.T/C.G	TACTCAACT	Ser->Ser
256G	M	66	1.83	N	SQ	IIIb	4	1	10	C.G/G.C	AAACTCCTT	Leu->Leu
937H	M	74	1.46	C	SQ	II	2	1	215	T.A/G.C	CTGGTGGTG	Val->Gly
845H	M	75	1.46	C	A	IIIa	3	0	215	T.A/G.C	CTGGTGGTG	Val->Gly
466J	F	76	0	C	A	IIIb	4	0	57	G.C/A.T	ACTCAGCAA	Gln->His
783J #	M	73	0	-	A	II	2	1	18	A.T/C.G	TACTCAACT	Ser->Ser
888C	M	65	0.73	N	A	II	2	1			Wild type	
968F	M	68	1.46	C	SQ	II	2	1			Wild type	
362C	M	66	1.46	C	SQ	II	2	1			Wild type	
499F	M	67	1.46	C	A	IIIa	3	0			Wild type	
849G	M	66	0	C	A	I	2	0			Wild type	
627H	M	65	5.84	C	SQ	I	2	0			Wild type	
664G	M	48	0	N	A	IIIa	2	2			Wild type	
830G	M	61	0	N	A	IIIb	4	0			Wild type	

M: Male; F: Female; T: Tumor factor; N: Node factor. * Total cigarettes ×10^4^. ** N: positive immunostaining in nucleus; C: positive immunostaining in cytoplasm; -: negative immunostaining. *** A: adenocarcinoma; SQ: squamous cell carcinoma. # One of 151 patients with Cx43 negative immunostaining in tumor tissues had Cx43 gene mutation.

## Data Availability

Dataset available on request from the authors.

## Supplementary Materials

The following supporting information can be downloaded at: https://www.mdpi.com/article/10.3390/medicina60101641/s1, Figure S1: Aberrant localization of mutant Cx43 protein in transfected CL-3 lung adenocarcinoma cells.
