# Supplementary material for: Mutations of the Cx43 Gene in Non-Small Cell Lung Cancer: Association with Aberrant Localization of Cx43 Protein Expression and Tumor Progression
Source: Medicina (Kaunas). 2024 Oct 7;60(10):1641. doi: 10.3390/medicina60101641 (PMC11509097; doi:10.3390/medicina60101641)
Supplement: Supplementary file 1 [file medicina-60-01641-s001.zip › medicina-3220921-supplementary.pdf]

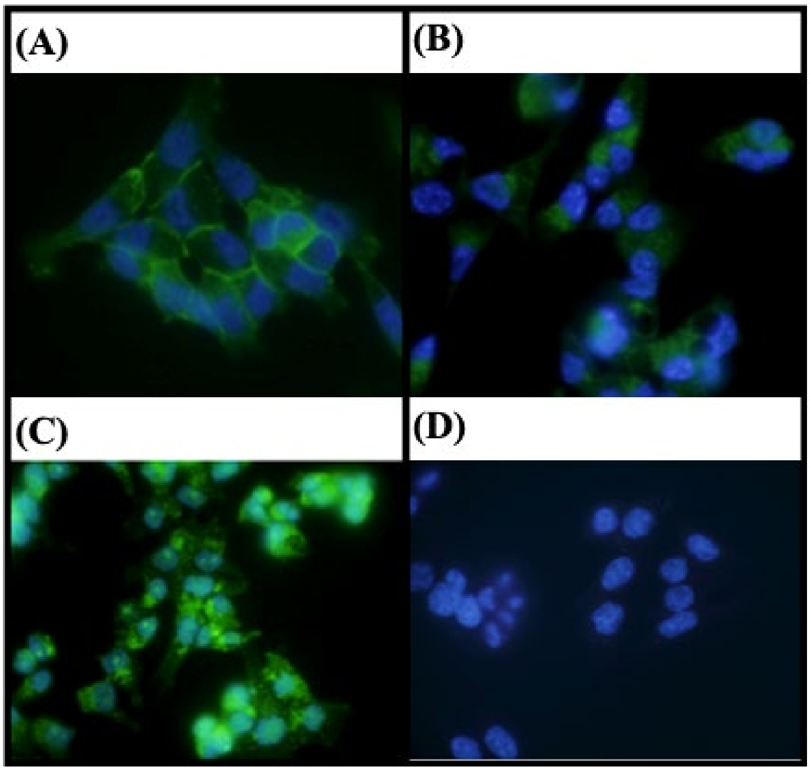

Figure S1. Aberrant localization of mutant Cx43 protein in transfected CL-3 lung adenocarcinoma cells. Representative immunofluorescence images showing Cx43 protein expression and localization in CL-3 lung adenocarcinoma cells transfected with different Cx43 constructs. (A) Wild-type Cx43. (B) Cx43 with codon 57 G/A mutation. (C) Cx43 with codon 10 C/G mutation. (D) Untransfected CL-3 cells (control). Cx43 protein is visualized in green, and cell nuclei are counterstained with DAPI (blue). Cx43, Connexin 43.
